# Application of Artificial Intelligence Techniques to Predict Survival in Kidney Transplantation: A Review

**DOI:** 10.3390/jcm9020572

**Published:** 2020-02-19

**Authors:** Covadonga Díez-Sanmartín, Antonio Sarasa Cabezuelo

**Affiliations:** Department of Computer Systems and Computing, School of Computer Science, Complutense University of Madrid, 28040 Madrid, Spain; mcdiez@ucm.es

**Keywords:** artificial intelligence, machine learning, survival, kidney transplantation

## Abstract

A key issue in the field of kidney transplants is the analysis of transplant recipients’ survival. By means of the information obtained from transplant patients, it is possible to analyse in which cases a transplant has a higher likelihood of success and the factors on which it will depend. In general, these analyses have been conducted by applying traditional statistical techniques, as the amount and variety of data available about kidney transplant processes were limited. However, two main changes have taken place in this field in the last decade. Firstly, the digitalisation of medical information through the use of electronic health records (EHRs), which store patients’ medical histories electronically. This facilitates automatic information processing through specialised software. Secondly, medical Big Data has provided access to vast amounts of data on medical processes. The information currently available on kidney transplants is huge and varied by comparison to that initially available for this kind of study. This new context has led to the use of other non-traditional techniques more suitable to conduct survival analyses in these new conditions. Specifically, this paper provides a review of the main machine learning methods and tools that are being used to conduct kidney transplant patient and graft survival analyses.

## 1. Introduction

In 2002, the Kidney Disease Outcomes Quality Initiative (K/DOQI) defined the term “chronic kidney disease” (CKD) and classified its seriousness levels [1]. CKD is a disorder that consists in the gradual loss of the kidney function. According to the figures given by the association of European kidney specialists, ERA-EDTA (European Renal Association—European Dialysis and Transplant Association) [2], it is estimated that 850 million people worldwide have some form of kidney disease. All CKD stages are associated with a higher risk of early death, cardiovascular morbidity and decreased quality of life. If not treated, CKD can advance and cause a kidney failure, until, at a given point, the kidneys cease to work. The only option for survival in end-stage renal disease (ESRD) is Renal Replacement Therapy (RRT), such as haemodialysis, peritoneal dialysis and kidney transplant. These treatments extend patients’ lives, but do not cure the disease.

Mortality among CKD patients who use an RRT therapy amounts to 8 to 9% of the total of dead CKD patients, where 2.8% of the mortality considered is due to patients who had received a transplant [3]. It is interesting to study the data available on patients who underwent kidney transplants to assess the success or failure of this therapy and compare it to the information from patients who underwent other types of RRT. This kind of comparative study is what is known as a survival analysis. Even though there are various approaches to this study, the current trend in the study of survival analysis in kidney transplant focuses on long-term renal graft survival [4,5,6], basically due to advances in Major Histocompatibility Complex (MHC) and immunosuppressive therapy (such as use of ciclosporin), which has improved short-term graft survival and reduced the acute renal graft rejection rates [7].

Traditionally, to conduct the survival analysis, a number of classic statistical terms are used, seeking to model the time that it takes for an event of interest to occur (e.g., the death of a transplant patient or the emergence of symptoms of transplant graft rejection) [8]. However, one limitation on the application of this kind of technique is the fact that the event of interest cannot always be fully observed due to the lack of available data on the RRTs performed. Thus, the most frequent form of data non-completeness in survival analyses is what is known as right-censoring [9]. The term “censoring” informally refers to situations where there is a loss of information regarding the variable of interest, that is, sometimes it cannot be observed, but what is observed is another variable known as the censoring variable. Right-censoring is a type of censoring that usually occurs for the following reasons [10]: an individual does not experience the event before the end of the study, a subject ceases to be monitored during the study period, the individual leaves the study due to their death (if the cause of their death is not the event of interest) and other reasons. The statistical techniques most frequently used to deal with censoring are, according to [11], the Kaplan-Meier estimator and Cox regression. Scientific literature includes many studies that apply these techniques to data pertaining to kidney transplant. For example, in [12] the rate of kidney failure after transplant in Europe between 1986 and 2015, and [13] studies the mortality due to a cerebrovascular accident in kidney transplant recipients using the data in the Taiwan National Health Insurance Research Database (NHIRD) from 2000 to 2011. The Kaplan-Meier estimator [14] is a statistical technique that makes it possible to estimate subjects’ survival function using as data only the time that they remain alive, and the function indicating censoring. As for Cox regression [15], it is a class of regression model that, in addition that using the survival time and the variable indicating censoring, uses additional data (regressor variables), such a sex, age, whether the subject is a smoker and others.

The statistical techniques that were initially used were adequate in a scenario in which the quantity and variety of the data available were limited. However, two events have occurred in the last decade that have changed this situation. The first one is the digitalisation of medical information, and specifically, the introduction of electronic health records (EHRs) that contain patients’ medical histories and facilitate computer processing of information. The second has been the Big Data [16,17] phenomenon, which is characterised by the availability of vast amounts of data in various formats, generated at an exponential speed. Thus, the exploitation of the information included in those data can assist decision-making (e.g., in medicine it can help to improve the doctors’ diagnoses [18,19,20] that are currently made on the basis of their experience with similar cases, such as the interpretation of medical images [21,22,23], among others). This phenomenon has also affected medicine, and made available vast amounts of data on various digital formats on the medical and healthcare activity of the patients who come to health centres or undergo a healthcare procedure (blood tests, X-rays, treatments, operations, organ transplants, among others). To optimally process this information under the new conditions, other analysis techniques are required, such as machine learning algorithms [24] in the field of artificial intelligence [25]. These algorithms make it possible to create models that can learn automatically and generate predictions from previous knowledge or experience on a specific topic, improving information processing with no need to explicitly program all the possible cases. In addition, in many cases, algorithms can improve their capacity by acquiring new experiences that refine and improve the system by providing more knowledge about the problem that they try to solve. Researchers in [26] give some reasons that makes the use of machine learning in processing tasks interesting, particularly in the field of biomedicine:

- They help to extract the factors considered by experts in their field of study when evaluating a situation or making decisions.
- They make it possible to find unknown functional relationships or properties among the entry data.
- They quickly adapt to changing environments with no need to redesign the system if the data are updated or replaced by other data.
- They can handle missing and noisy data.
- They make it possible to find relations and correlations among large amounts of data, and to generate solutions with a high degree of accuracy.

The purpose of this paper is to provide a narrative review of the main machine learning techniques used to conduct the survival analysis for kidney transplantation.

For this review, searches were carried out in the following free search engines: PubMed [27], Science Direct [28] and DBLP (Digital Bibliography & Library Project) that is a computer science bibliography website [29]. Luck et al [30] was found in the bibliography when Tapak et al was being reviewed [31].

The search terms were [kidney, transplant, graft, graft failure, survival analysis, survival, machine learning, neural network, deep learning, random forest, random survival forest, classification and regression tree, decision tree, C5.0, J48, survival decision tree, svm, bagging, support vector machine, multilayer perceptron, naïve Bayes, k-nearest neighbour], comprising all the literature between January 2016 and May 2019.

The papers that met the following criterion were selected: use of machine learning techniques to solve problems in the survival analysis of kidney transplant patients, written in English. This exploratory search resulted in the selection of nine papers.

As can be seen in Table 1, the papers reviewed are very heterogeneous in terms of the size of the population analysed as well as in terms of the methods used. All methods mentioned are explained in the Section 2.1 and in Appendix B.

The paper is structured as follows. Section 2 presents the review conducted in two different subsections. Section 2.1 shows the various machine learning techniques being used to conduct survival analyses in the area of kidney transplants. Section 2.2 describes the situations in which the techniques described are being applied. The papers analysed are then discussed in Section 3, and, finally, the future directions and conclusions are given in Section 4 and Section 5.

In the Appendix A, a more detailed explanation of the machine learning techniques most used in the reviewed articles is provided and in Appendix B the rest of methods mentioned is explained.

## 2. Results

### 2.1. Machine Learning Techniques

This section presents the first result of the review carried out. These are the machine learning algorithms used in the analysis of survival in kidney transplant. The use of decision trees, ensemble methods, neural networks and support vector machines is described.

#### 2.1.1. Decision Trees

**Figure 1:**
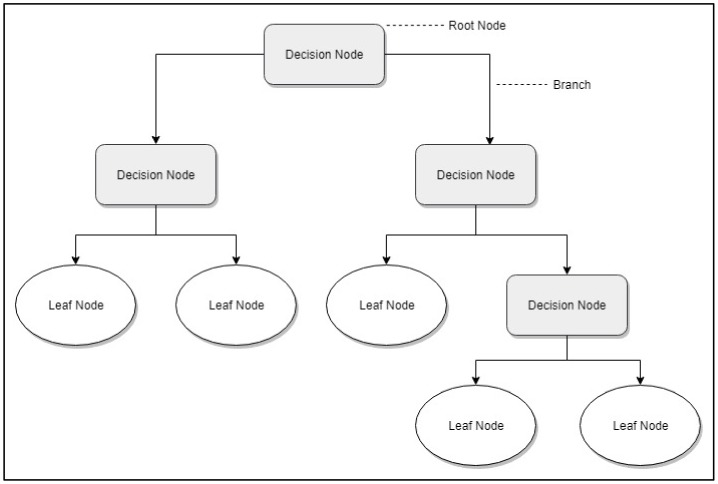
Topology of decision tree.

Decision trees [38] are a segmentation method that tries to achieve a classification into homogeneous groups of the observed sample, gradually segmenting them in accordance with the variable of interest or segmentation variable. To do so, a division process is carried out in tree form, where nodes represent the features of the sample to be classified, and each tree branch represents a possible value of that feature (Figure 1). A sample subject is allocated to a specific segment by selecting the features that best discriminate and by building a decision rule that makes it possible to select the best possible division at each of the division process levels. This feature will define the first division of the sample into two segments. Then, the previously created segments are segmented again, and division continues successively until the process end, using a stop criterion previously established or voluntarily halting the process [39]. A more detailed explanation of the decision trees can be found in Appendix A.

According to the review carried out, in the survival analysis, there are several types of decision trees used like CART (Classification And Regression Trees) [40,41,42,43,44], C5.0 [36], J48 [34] and the survival decision tree model. CART [45] is a method that works with all types of variables, with no need to make continuous features discrete, so that the classification is used when the variable of interest is categorical and regression is used in the case of a continuous variable. C5.0 algorithm [46,47] is the extension of C4.5 algorithm. C5.0 improves the speed, memory and the efficiency of its predecessor [48]. J48 algorithm [47] is an open source implementation of the C4.5 decision tree algorithm.

As for the survival decision tree, it is a prediction model composed of decision trees [49] to estimate the survival functions [50] used to represent the probability that a subject will survival beyond a pre-specified point in time. The main difference between decision trees and survival decision tree is the selection of the segmentation criterion [51]: rather than using the entropy index (it is a statistic that measures the degree of homogeneity of the data from a segmentation given by the machine learning algorithm) or the Gini index (it is a statistic that measures the degree or probability that a variable is misclassified when taken randomly) [52], as conventional decision trees do, survival decision trees can use, e.g., the survival analysis statistic [50]. The survival decision tree also considers the interactions among the explanatory variables and can process censored data using a tree structure.

#### 2.1.2. Ensemble Methods

Ensemble methods are a machine learning technique in which multiple models are trained (or estimated in the usual statistical language), in order to combine their predictions and thus achieve a more accurate result. According to the papers reviewed, two ensemble methods are being used in survival analysis: bagging and the random forest method.

The ensemble method known as bagging or bootstrap aggregation [53] is to create different models by using random samples with substitution, to later combine the results. This model reduces the variance of the base models being used. Bagging is usually performed on the basis of decision trees. In survival analysis, bagging is performed on the basis of survival decision trees [53,54].

**Figure 2:**
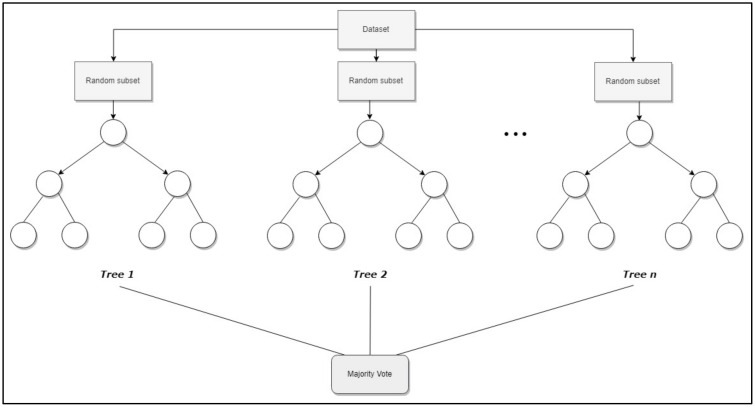
Topology of Random forest.

Regarding random forest (RF) [55], it is an ensemble method that adds more randomness to bagging, because, in addition to randomly selecting observations, it also randomly selects subsets of the explanatory variables. The algorithm constructs a large number of trees (a “forest”), which are trained, and finally a prediction based on the majority vote principle is obtained (Figure 2), i.e., “after a large number of trees are generated, they vote for the most popular class” (see Section 1 in [55]). A more detailed explanation of RF can be found in Appendix A.

The standard approach to analyse survival data using random forest is known as random survival forest (RSF) [56], which extends the RF method. RSF is a method for the analysis of right-censored survival data. The algorithm combines bootstrap techniques to select the observations and the explanatory variables with other statistical techniques to generate the trees and finally to estimate the function of the cumulative risk rate in presence of censored data.

#### 2.1.3. Artificial Neural Networks

Artificial Neural networks [57] (ANNs) are a regression or classification model inspired in biological neural networks. The structure of a neural network is composed of nodes, layers and synapses. A node or neuron is the basic element of a computation. It receives the entry data from an external data source or another node. Every entry has an associated synaptic weight that is modified through the learning process. A neuron consists of a set of entries, a propagation rule (which determines the potential result of the neuron’s interaction with its neighbouring nodes), an activation function (which determines the neuron current activation status) and an exit function (which yields the neuron’s exit value). These functions depend on the neural network used. The layers are structural units that group nodes. Nodes are interconnected by synapses with an associated weight (synaptic weight). The network’s behaviour is determined by the structure of the synaptic connections.

According to our review, one of the neural network architectures most frequently used in the study of survival analyses in the area of kidney transplants is the multilayer perceptron (MLP) [58]. MLP is a neural network that contains one or more hidden layers and uses the backpropagation (BP) algorithm [59] to train. This type of network is used to directly model the survival function [30] as well as to classify graft and patient survival [34,35]. In Appendix A, more detailed information on the ANN can be found.

#### 2.1.4. Support Vector Machines

**Figure 3:**
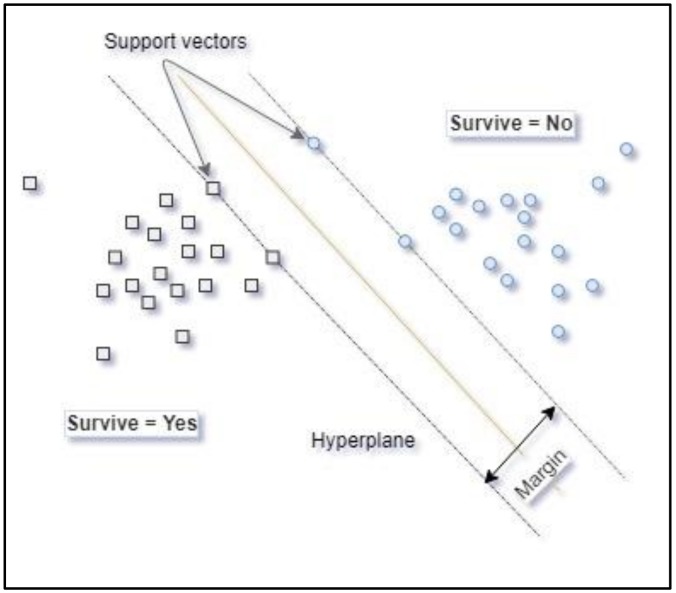
Graphic representation of the SVM hyperplane.

The machine learning technique known as support vector machine [60] (SVM) uses entry data represented in a features space that can have multiple dimensions and generates an optimal function for separation of the values of the variable to be modelled. If the population is separable or quasi-separable (presence of noise), a linear function that can separate the data in the original space of the entry samples is used. When the population is not linearly separable, a kernel function is used to transform the original entry data and take them to a new space of features where the hyperplane can achieve linear separation of the classes in the variable to be modelled. Figure 3 shows that the separation hyperplane is equidistant from the closest observations in each class to constitute a maximum margin on either side of the hyperplane. The support vectors are the observations on the border of these margins. A more detailed explanation of SVM can be found in Appendix A.

In the literature reviewed, SVM are used in survival analysis as a classification technique without modelling the survival function [34,35].

### 2.2. Application of Machine Learning Algorithms

This section presents the result of the review carried out. The ways in which the machine learning algorithms described in the previous section are applied to the survival problem are described. The papers examined show two different approaches: (i) classification of transplant patients depending on whether they survive and (ii) modelling of the survival function to estimate patients’ survival time. These two approaches are described below.

#### 2.2.1. Classification of Patient Survival

This approach is intended to predict whether transplant patients or grafts will survive for a given period of time after the reception of the transplant or predict survival in terms of time ranges or risk classes. The authors of [34] proposed a new method to predict five-year kidney graft survival, which combines a hybrid feature selection function to select the minimum number of features that make the analysis most accurate, and the K-nearest neighbour (KNN) algorithm (more information is provided in Appendix B) to classify input data, such as survival and non-survival. The hybrid feature selection function combines the information gain criterion with the naïve Bayes classifier (see Appendix B for more information), selecting the minimum number of features that generate the highest accuracy. In this study, censoring is measured in days in the graft time variable. The method proposed was experimentally evaluated, proving that it exceeds, in terms of accuracy (cross-validation) and F-measure, techniques such as the J48 algorithm, naïve Bayes, multilayer perceptron ANN, RF and SVM, as it achieves maximum accuracy and the F measurement with minimum errors. The accuracy value achieved in experiments shows that the KNN indicators improves its classification capacity when combined with a feature selection function rather than without it.

The authors of [35] evaluated three machine learning techniques to predict kidney transplant recipients’ five-year survival: multilayer perceptron of artificial neural network, SVM and logistic regression (LR). The authors did not mention any specific treatment for censored data. The criteria used to compare the results obtained with each technique are: sensitivity (ratio of surviving subjects which the model has classified as surviving), specificity (which indicates the ratio of subjects predicted as graft rejections have rejected the graft), accuracy (number of correct predictions carried out by the model from among all the predictions made) and AUROC (Area Under the Receiver Operating Curve). The results of the study show that SVM is the best technique at 90.4% accuracy, 98.2% sensitivity, 49.6% specificity and 86.5% AUROC. It is followed by MLP, at 85.9% accuracy, 97.3% sensitivity, 26.1% specificity and 76.9% AUROC. LR would come last, with an accuracy, sensitivity, specificity and AUROC of 84.7%, 97.5%, 17.4% and 77.4%, respectively. Thus, the study experimentally proves that for the data analysed, two machine learning techniques surpass the logistic regression technique whose discriminating capacity has been established in the statistics literature.

The authors in [31] also approached survival from a binary classification, i.e., transplant success or failure. Two predictive techniques are used: a neural network (an MLP) and a logistic regression model. As in the previous case, predictive power is measured on the basis of the accuracy, sensitivity, specificity, and AUROC metrics, obtaining values of 75%, 91%, 74% and 88%, respectively, for MLP and 55%, 91%, 51% and 75% for LR.

The authors in [36] evaluated the C5.0, multilayer perceptron of artificial neural network, and CART to predict kidney transplant survival before the transplant. The authors do not mention any specific treatment for censored data. Accuracy is considered to compare the results obtained with each technique. The most accurate model is C5.0 (96.77%), followed by CART (83.7%) and the neural network at 79.5%. This study shows that, with the data analysed, survival prediction is more accurate when tree-based algorithms rather than neural network-based algorithms are used.

The authors in [37] used a multinomial classification to classify the kidney graft’s survival degree into three risk classes: high risk (0 to 3 years), medium risk (3 to 7 years) and low risk (more than 7 years). To do so, expert judgement was combined with information derived from the combination of a feature selection framework function, machine learning algorithms: SVM, ANN and bootstrap forest (in an RF approach) [59,60,61], and the elastic net variable regularisation and selection technique [62]. Bayesian belief networks (see Appendix B for more information) were also used to identify non-linear relations and interactions between the explanatory factors and risk levels for kidney graft survival. The advantages of this model are the possibility of modelling the interaction between a large number of variables and use of a variable selection methodology based on objective metrics that enrich expert judgement. The weak point of the study is its failure to evaluate the predictive power of the approach proposed with respect to other classification techniques.

#### 2.2.2. Modelling the Patient Survival Function

This approach tackles the problem of estimating the distribution function for the time between transplant and graft failure considering data censoring. In [7], authors focused on patient survival after a kidney transplant and evaluated the survival decision tree technique with respect to a decision tree and a Cox regression model. The study used as explanatory variables the 3-month serum creatinine level post-transplant, which is known to offer a high degree of discriminating capacity in the field of kidney transplants. The result obtained was that the survival decision tree offers greater predictive power than conventional decision trees and Cox regression models. The study also examined other tree-based models: bagging and RF. However, it provides no results on the performance of the models examined to compare the different techniques.

The authors in [32] describes an ensemble method that combines the RSF model with conditional inference trees (RF constructed on a non-parametric class of decision trees, see more information in Appendix B) [53,63] with the Cox’s proportional hazards model to create a survival prediction model for kidney transplant patients and identify the predictive variables. To do so, the importance variable based on the Breiman-Cutler method [64] for RSF is used to order the variables in terms of their importance in the model’s prediction. The result is that the best variable is the graft recipient’s age. Then, the data were segmented considering this variable, using decision trees that generate two cohort groups (cohort 1: subjects aged 50 or younger, and cohort 2: subjects aged 51 or older). The algorithm selects the model that offers the best results for each cohort (RSF with conditional inference trees or Cox’s proportional hazards). In the case of cohort 1, more accurate results were obtained by using RSF models with conditional inference trees as base learners, while, for cohort 2, the best results were obtained on the basis of the Cox’s proportional hazards model. The advantage found on the basis of the results presented by the authors is that the model obtained improves the estimated power of the Estimated Post-Transplant Survival (EPTS), a measure used to allocate some kidneys in the United States kidney allocation system [65] (0.724 against 0.697), proposing a new graft survival methodology.

The authors of [33] developed an online tool to evaluate whether kidney offers from marginal donors (i.e., older people or people with medical comorbidities) provided candidates with a substantial survival benefit. In this paper, the authors analysed survival prediction after transplant and for patients on the waiting list to receive a kidney. In the first case, they used the RSF algorithm combined with Kidney Donor Profile Index (KDPI, see Appendix B for more information) and the EPTS, and compared it to the Kidney Donor Risk Index (KDRI, more information of this estimator could be found in Appendix B) model in terms of the C-index statistic. In the second case, the authors predicted waitlist survival by EPTS using a Weibull model (see Appendix B for more information) estimating directly the survival function of the patients. The result of the evaluation yielded a 0.637 index for the RSF algorithm, which is slightly higher than KDRI model, which yields an approximate C-index value of 0.6.

The authors of [30] evaluated a multilayer perceptron of an artificial neural network that taking into account two kinds of information loss, the presence of ties and the censored nature of the dataset. Its performance was compared to the use of Cox’s regression method in terms of the C-index statistic and AUROC, modified to consider right-censored data. A 0.6550 C-index was obtained for the former model, and a 0.6504 C-index was obtained for the latter model. Thus, the 0.0046 difference in the C-index statistic was not statistically significant, and it could not be concluded that there was a gain in the predictive power of the model proposed. However, the main advantage of this study was that it considered the existence of censure in MLP configuration, and ties in terms of survival time using Efron’s technique [66] were considered.

## 3. Discussion

By using electronic health records in hospitals, the volume and complexity of the data connected to each patient substantially increases. For this reason, the need arises to replace traditional quantitative techniques by artificial intelligence algorithms that can process mass data. In the context of survival analyses in the area of kidney transplants, the review has found that some of the techniques most frequently used in the recent past have been neural networks, survival decision trees, random forest and support vector machines, among others.

According to the literature reviewed, the problem of survival analysis is tackled from two different approaches: as a classification problem or as a problem of survival function modelling.

The first approach treats it as a classification problem, focusing on predicting whether patients or grafts survive a specific period of time. Table 2 shows the papers that classify patient and graft survival. The first three papers compared the predictive power of several machine learning models, the first of which being the one with the highest predictive power with an SVM model. The last two papers proposed a hybrid model, focusing on the selection of the features that offered the highest predictive capacity for the algorithm used.

In Table 2, the classifier’s performance is measured in terms of accuracy and sensitivity. The sensitivity is a statistic that measures the probability of correctly classifying patients who survive transplantation according to how survival was classified in each of the studies cited in this table. The accuracy is a statistic that takes values between 0 and 100, with 0 being the absence of precision and 100 being the maximum precision.

In Table 2, the benchmark techniques used are machine learning models known in the context of classification [67] (fourth paper in the table) or a logistic regression (first and second papers). In both cases, the model proposed provided better results than the benchmark.

The second approach focuses on predicting transplant patient survival periods, that is, the survival function. Table 3 shows the papers that follow this approach. As can be seen, there are two types of study: those that uses a neural network (the third paper) and those that use tree-based models (the other papers). As regards the latter, in all cases random forest was used; however, only two of them used a type of RF adapted to consider the presence of data censure, random survival forest [56].

In Table 3, the benchmark is the classic statistical technique, Cox’s regression (first three papers), and the EPTS and KDPI metrics (last paper). The methods proposed outperform the benchmarks in terms of C-Index. C-Index is a statistic that measures the goodness of fit of probability models. It is commonly used in survival models as it is adapted to take into account censored data. This statistic takes values between 0 and 1, the closer to 1 the better the goodness of fit obtained.

Finally, Table 4 provides a summary of the factors that influence transplant patient or graft survival. To do so, the two most highly ranked factors in each of the papers analysed have been selected (two have been selected because Shahmoradi et al., 2016 only has two). Five out of the eight papers mention the factors that influence survival [7,31,32,35,36,37].

Other factors influencing survival that was mentioned in the papers review are: hypertension, smoking, a history of viral hepatitis B and C, cerebral and peripheral vascular disease, recipient ethnicity category or recipient HCV status, among others.

There are also several factors that influence transplant outcomes that has not been mentioned like, for example, retransplantation [68,69,70], infection or malignancy post transplant [71].

## 4. Future Directions

At present, machine learning techniques are seen, in general, by both researchers and practitioners as black boxes. This leads to the natural interest of how to make these algorithms more explainable. In particular, it would be interesting to be able to determine the contribution of each of the explanatory variables, or even, of the possible interactions between them in the models, trying to achieve the highest possible level of explanation, for example, as in the traditional regression models. Today, as seen in the review, this cannot be determined directly, but only from the final output of the model (C-Index, AUC, sensitivity, accuracy, among others).

## 5. Conclusions

In the context of survival analyses in the area of kidney transplants, the review has found that some of the techniques most frequently used in the recent past have been neural networks, survival decision trees, random forest and support vector machines. The review conducted also shows that machine learning techniques are being mainly used for two purposes. Firstly, as a mechanism to model the survival function, and secondly, as mechanisms for patient or graft classification in terms of transplant survival or non-survival.

The first goal involves predicting transplant patient or graft survival periods, that is, estimating the survival function. The most widely used techniques are tree-based, adapted to consider the presence of data censure, namely survival decision trees and random survival forest. The cases examined show that the predictive power of the models proposed surpassed one of the classic techniques most frequently used in survival analysis, namely Cox’s regression.

According to the literature under review, there is a certain trend to use some machine learning algorithms, such as, for example, the multilayer perceptron neural network or support vector machine approaches, focusing on transplant patients’ or grafts’ classification capacity, that is, whether they survive or not, rather than estimate their survival time. This can be due to, among other reasons, the limitations of these techniques due to their not being sufficiently adapted to the censored data scheme which is usually present in the study, the short-term observation windows, limited knowledge of the factors that influence transplant patients’ or grafts’ survival, or a potential high disproportion between positive cases (patients or grafts in whom the event of interest is observed) and the negative cases (survivors at the end of the study and censored cases).

As was previously discussed, one of the most interesting features of machine learning algorithms is their flexibility and their capacity to adapt to the problem of interest. In this respect, one of the strategies that is receiving the most attention in recent literature is the use of ensembles of machine learning algorithms, that is, the combination of various algorithms in order to make maximum use of the potential of each model to achieve better predictions.

In the recent literature of machine learning models, it is not possible to find unanimity on which is the best algorithm under any data structure, that is, an omnibus algorithm that is above the others. On the contrary, given the nature of these algorithms, which are highly dependent on both the structure and the quality of the input data, in certain circumstances one type of algorithm may outperform others or vice versa. An example that synthesises this idea can be observed by contrasting the results presented in Table 2, where the accuracy ratios thrown by the SVM in article [39] and article [38] are not even similar.

## Figures and Tables

**Table 1:** Papers analysed in this study.

Authors	Data Source	Population	Methods Used
Yoo et al., 2017 [7]	Three centers in Korea (1997–2012)	3117	Survival decision tree, bagging and random forest
Mark et al., 2019 [32]	United Network for Organ Sharing (UNOS) (1987–2014)	100,000	Combines predictions from random survival forest constructed from conditional inference trees with a Cox proportional hazards model
Bae et al., 2019 [33]	Organ Procurement and Transplantation Network (OPTN) (2005–2016)	120,818 kidney recipients and 376,272 candidates on the waiting list	Combinations of The Kidney Donor Profile Index and the Estimated Post Transplant Survival score using random survival forest, and waitlist survival by Estimated Post Transplant Survival score using Weibull regressions
Atallah et al., 2019 [34]	Urology and Nephrology Center, Mansoura, Egypt (1976–2017)	2728	Merges information gain with Naïve Bayes and k-nearest neighbour
Nematollahi et al., 2017 [35]	Nemazee Hospital, Shiraz, southern Iran (2008–2012)	717	Artificial neural network and support vector machines
Tapak et al, 2017 [31]	Ekbatan or Besaat hospitals, Iran (1994–2011)	378	Artificial neuronal networks
Shahmoradi et al., 2016 [36]	Sina Hospital Urology Research Center, Iran (2007–2013)	513	C5.0, artificial neural networks and classification and regression trees
Luck et al., 2017 [30]	Scientific Registry of Transplant Recipients (2000–2014)	131,709	Artificial neural network taking into account two kinds of information loss, the presence of ties and the presence of censoring.
Topuz et al., 2018 [37]	UNOS (2004–2015)	31,207	Feature selection with support vector machine, artificial neural network and bootstrap to construct a Bayesian belief network

**Table 2:** Classification of patient survival.

Authors	Method Used	Benchmark	Best Performance	Accuracy	Sensitivity
Nematollahi et al., 2017 [35]	ANN and SVM	Logistic regression	SVM	90.40	98.20
Tapak et al, 2017 [31]	ANN	Logistic regression	ANN	75	91
Shahmoradi et al., 2016 [36]	C5.0, ANN and CART	N/A	C5.0	87.21	90.85
Atallah et al., 2019 [34]	Merges information gain with naïve Bayes and K-NN	J48, Naïve Bayes, ANN, RF, SVM, KNN	Proposed method	80.77	80.40
Topuz et al., 2018 [37]	Feature selection with SVM, ANN and bootstrap to construct a Bayesian Belief Network	N/A	Proposed method	68.40	41.00

**Table 3:** Modelling the patient survival function.

Authors	Method Used	Benchmark	Best Performance	C-Index
Yoo et al., 2017 [7]	Survival decision tree, bagging, and RF	Decision tree and Cox regression	Survival decision tree	0.80
Mark et al., 2019 [32]	Combines predictions from RSF constructed from conditional inference trees with a Cox proportional hazards model	EPTS, Cox model, random forest	Proposed method	0.724
Luck et al., 2017 [30]	Artificial neural network taking into account two kinds of information loss, the presence of ties and the presence of censoring.	Traditional Cox model using Efron’s method	Proposed method	0.6550
Bae et al., 2019 [33]	Combinations of KDPI and EPTS using RSF	KDRI	Proposed method	0.637

**Table 4:** Main factors influencing survival.

Recipient/Donor Factors
The 3-month serum creatinine level post-transplant
Recipient age
Kidney cold ischemic time
Donor age
Discharge time creatinine
Body mass index
Pre-transplant dialysis
Recipient functional status at registration
Recipient diabetes at registration

**Figure A1:**
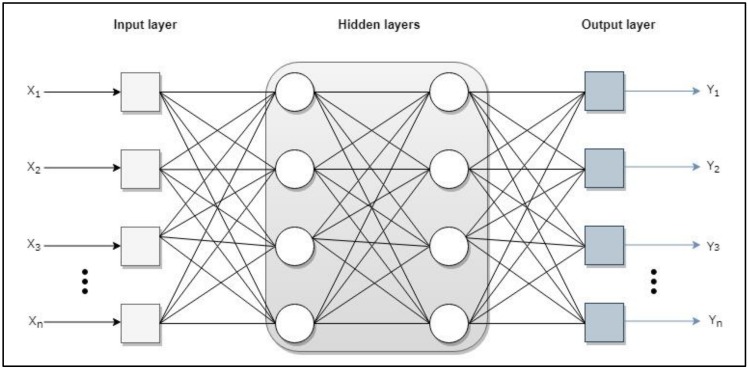
Topology of Multi-layer network.

## Appendix A

This appendix explains in more detail the machine learning techniques most used in the reviewed articles.

### Decision Tree

The decision tree [72] is an algorithm that uses a tree-shaped structure to model the relationships between the characteristics. This structure begins in a trunk known as root node and is divided into branches. Decisions are made in each of these divisions or decision nodes. Finally, it ends up reaching the leaf nodes where the data is homogeneous enough not to continue dividing them further.

The division of the tree is usually done by identifying the feature that will star in that division. In most cases, splitting functions are univariate, that is, an internal node is divided according to the value of a single feature. There are several univariate criteria, such as criteria based on impurities, binary or information theory [72]. Some of the most used criteria are those in which the purity of the data is checked, that is, the fact that the data contains a single class. There are different measures of purity to identify the best candidate for division of the decision tree. Some of the most used are entropy and the Gini Index [52,73].

The tree growth phase can continue until a stop criterion is met, for example, that the division criterion is not better than a given threshold or that the maximum tree depth is reached [72].

Some notable advantages are that they require less effort in preparing the data in the preprocessing phase, which is intuitive and easy to explain.

One of the main disadvantages is that decision trees are that they are very sensitive to data structure, attributes that do not provide relevant information and noise [74].

### Random Forest

The random forest (RF) method [55] works with sets of decision trees combining bagging principles [75] with random selection of features to add additional diversity to the decision trees. Once the trees are generated, they work as a whole. Each tree in the RF calculates a class prediction, with the class with the most votes (majority vote) being the model prediction. The goal is that many uncorrelated trees that work together outperform any of the individual trees that make it up.

RF offers some advantages over decision trees such as reducing the possibility of over-fitting, especially when the tree is of a particular dimension or can be used in data with a large number of features or samples, among others.

The decision trees are one of the “black-box” type of algorithm, this is that it is not easily interpretable because it does not have a simple display to see the model as in the case of the decision trees. In addition, it may require work and experience to adjust the parameters that optimise the algorithm configuration.

### Artificial Neural Networks

An artificial neural network [76] (ANN) models the relationship between a set of input signals and an output using a model that has been made based on how it is understood that a brain works. That is, how it reacts to stimuli using a network of interconnected cells called neurons to create a massive parallel processor. That is why it is said that ANNs use a network of artificial neurons or nodes to solve learning problems.

This type of algorithm is known as black box because it is not possible to know the model it uses.

(1) $$y\left(x\right)\;=f\left(\sum_{i=1}^{n}w_{i}x_{i}\right)$$

Equation (1) explains how a single artificial neuron works. A relationship between input signals (variables x) and an output signal (dependent variable y) is defined. Each of the inputs received receives a weight (w_i_) according to its importance. These are added together and the signal obtained is passed through an activation function f.

From the basic scheme of operation of a single neuron, different layers can be concatenated to end up obtaining a more complex network that represents a set of data from which it would like to end up obtaining a model. In general, neurons are usually grouped into structural units called layers. Within a layer, neurons are usually of the same type and there may be different numbers of neurons. Three types of layers can be distinguished (Figure A1):

- Input layer: receive data or signals from the environment.
- Output layer: provide the network response to input stimuli.
- Hidden layer: they do not receive or provide information to the environment (internal processing of the net).

According to the number of layers, neural networks can be classified into:

- Single-layer network. Only one input layer related to the output layer.
- Multi-layer network (MLP). One or more layers are added between the inputs and outputs, as can be seen in Figure A1. The MLP is a neural network that contains one or more layers of hidden neurons and uses the backpropagation (BP) algorithm [57] to train. This algorithm consists of two phases: feed-forward and feed-back propagation. In the feed-forward phase, the network output is calculated with all fixed weighting values while the input vector is entered from the input layer to the output one through the hidden layers. In the feed-back propagation phase, the error signal is calculated by subtracting the network output value from the expected output value. An error signal originates from an output neuron and spreads backwards layer by layer through the network [76]. It then propagates through the hidden layers to the input layer so that the weighting values are corrected. These two phases are repeated and the learning process is recycled to generate a better approach to the output. The learning process ends when the stop condition is met.

A neural network with multiple hidden layers is known as a Deep Neural Network (DNN) and the practice of training a network of this style is often called Deep learning.

Some of the characteristics of neural networks are that they can handle noise even in the presence of incomplete or distorted data and are fault tolerant since they have the information distributed among their neurons, thereby achieving some redundancy. Finally, neural networks act as a black box and cannot determine how information is processed internally.

### Support Vector Machine

A Support Vector Machine (SVM) [77] can be imagined as a surface that creates a division between data points represented in a multidimensional way that represents their classes and their characteristics. The objective of an SVM is to create a flat boundary known as a hyperplane that divides the space to create homogeneous partitions on each side of it.

The complexity of the SVM algorithm lies in the fact of identifying a line that separates the two classes. The algorithm looks for the hyperplane with maximum margin, that is, the hyperplane that maximises the distance to the data points closest to the classes.

This algorithm works efficiently with noisy data and with non-linear data. Some disadvantages of this technique are that the proper choice of kernel function is a difficult task and that it only supports numerical variables. The kernel function is a function that transforms the space of characteristics into another space, generally of greater dimension [77].

## Appendix B

This appendix explains the techniques or statistics that is used in some reviewed articles.

### Bayesian Belief Networks

A Bayesian belief networks (BBN) [78] is a probabilistic graphical model that make predictions based on a priori assumptions.

### Conditional Inference Trees

Conditional inference tree [63] is a type of regression tree that use statistical inference in its decision rules.

### Estimated Post-Transplant Survival

Estimated Post-Transplant Survival (EPTS) score [65] is a measure used to allocate some kidneys in the United States kidney allocation system.

### Information Gain

Information gain [71] is a measure for deciding the relevance of an attribute. In [34] this measure was used for feature selection, by evaluating the gain of each variable in the context of survival or not survival.

### K-Nearest Neighbours

K-nearest neighbours (KNN) [79] is a model that classifies each unlabelled sample by the majority label of its nearest neighbours.

### Kidney Donor Risk Index

Kidney Donor Risk Index (KDRI) is an estimator of the relative risk of post-transplant kidney graft failure model [80].

### Kidney Donor Profile Index

The Kidney Donor Profile Index (KDPI) [80] is a measure that summarises the quality of deceased donor kidneys relative to other recovered kidneys as a single figure.

### Naïve Bayes

Naïve Bayes is a probabilistic classifier based on Bayes’ theorem [81].

### Weibull Model

Weibull model [82] is one of the most popular parametric regression model [83]. It provides estimate of baseline hazard function and coefficients for covariates.
